# Assessment of Corneal Densitometry in Rheumatoid Arthritis Patients

**DOI:** 10.4274/tjo.89577

**Published:** 2017-06-01

**Authors:** Mustafa Alpaslan Anayol, Başak Bostancı, Mehmet Ali Şekeroğlu, Mert Şimşek, Süleyman Günaydın, Pelin Yılmazbaş

**Affiliations:** 1 Ulucanlar Eye Training and Research Hospital, Ophthalmology Clinic, Ankara, Turkey; 2 Keçiören Dünyagöz Hospital, Ophthalmology Clinic, Ankara, Turkey; 3 Kırıkhan State Hospital, Ophthalmology Clinic, Hatay, Turkey

**Keywords:** Corneal densitometry, Rheumatoid Arthritis, corneal topography

## Abstract

**Objectives:**

To evaluate corneal densitometry and anterior segment parameters of rheumatoid arthritis (RA) patients and compare these results with those of age-matched healthy control subjects.

**Materials and Methods:**

Anterior segment parameters and corneal densitometry of patients with RA and healthy control subjects were assessed by Scheimpflug corneal topography. For densitometry analysis, the 12-mm diameter area of the cornea was subdivided into four concentric radial zones and anterior, central, and posterior layers based on corneal depth. Right eyes of subjects were used for statistical analysis.

**Results:**

Twenty-three consecutive patients with RA and 22 healthy control subjects were included in the study. There was no significant difference with regard to age (p=0.487) or gender (p=0.514). When anterior segment parameters of both groups were compared, no significant difference was found (p>0.05). Total corneal densitometry values were statistically higher in the RA group (p=0.030). In addition, when subdivisions of the cornea were evaluated, higher densitometry values were found in the RA group in 0-2 and 2-6 mm radial zones both in the anterior and total depth (p=0.001, p=0.003 for the 0-2 mm zone and p=0.002, p=0.009 for the 2-6 mm zone). Corneal densitometry measurement was not correlated with central corneal thickness or simulated keratometry value in RA patients or healthy control subjects.

**Conclusion:**

The corneal densitometry values were higher in RA patients when compared to healthy control subjects, even if they had clinically clear corneas. Corneal densitometry as an objective measure of corneal clarity warrants further studies in order to ascertain its clinical relevance in RA patients.

## INTRODUCTION

Rheumatoid arthritis (RA) is a chronic inflammatory autoimmune disorder characterized by synovial joint involvement and extra-articular manifestations ranging from subcutaneous nodules to pulmonary, cardiovascular, cutaneous, and neurological involvement.^1,2^ The ocular surface is frequently affected and dry eye syndrome is the most common ophthalmic manifestation, followed by scleritis, episcleritis, anterior uveitis, and retinal vasculitis.^3,4,5,6^ Scleromalacia perforans and peripheral ulcerative keratopathy are other rare but frightening ocular complications. Rheumatoid arthritis is known to be the most common autoimmune disease to affect the cornea.^7,8,9,10,11^

Scheimpflug imaging is a useful tool for evaluating the cornea. The Pentacam^®^ HR (Oculus, Inc., Wetzlar, Germany) enables investigators to image both anterior and posterior corneal surfaces, providing a full pachymetry map. In addition, it is also possible to measure the amount of backscatter light for evaluating densitometry of different regions of the cornea with the new add-on software program.

Because anatomical regularity of the collagen fibrils, integrity of connective tissue, and balanced keratocyte components play an important role in corneal clarity, it can be hypothesized that corneal densitometry may be altered in the presence of a systemic inflammatory disease, even in the absence of any corneal haze or scar.^12^ The purpose of the present study was to evaluate anterior segment parameters and corneal densitometry in RA patients with clinically clear corneas and to compare these results with those of healthy control subjects.

## MATERIALS AND METHODS

This prospective controlled clinical trial was conducted at Ulucanlar Eye Training and Research Hospital. The study followed the tenets of the Declaration of Helsinki and was approved by the local ethics committee. The study included 23 consecutive RA patients and an age-matched control group of 22 healthy individuals. RA diagnosis and classification were based on previously published data and the patients with mild disease without any joint deformity were recruited for the study.^13^ All patients underwent ophthalmic examination including assessment of best-corrected visual acuity, intraocular pressure, slit-lamp examination, and fundoscopy prior to Scheimpflug imaging. Patients with corneal opacity; severe dry eye; glaucoma; any inflammatory ocular disorder or infection including blepharitis, conjunctivitis, meibomitis and dacryocystitis; central or peripheral thinning evident in slit-lamp examination; history of ocular surgery, trauma, or contact lens use; and patients using any topical medication other than artificial tears were excluded from the study.

Corneal power, corneal thickness, and corneal volume measurements were performed by Pentacam^®^ HR. Corneal densitometry analysis, provided as an add-on to the standard software of the Pentacam^®^ HR, was used for densitometry assessment. Using the 25 scan settings, the rotating system allowed corneal scans from 0 to 180 degrees, each photograph displaying the cornea at a specific angle.

Measurements were performed in the same clinical assessment room, using the black shield supplied by the company. In order to minimize the effect of diurnal changes in corneal hydration, all measurements were performed within the same time interval of the day (between 10 and 12 AM). The automatic release mode was used to determine when correct focus and alignment with the corneal apex had been achieved in order to reduce operator-dependent variables which may be associated with manual scanning. The output was expressed in gray-scale units. A maximum light scatter of 100 was defined for minimum transparency (completely opaque cornea), and minimum light scatter of 0 as maximum transparency (no clouding).

For analysis, the 12-mm diameter of the cornea was subdivided into four radial zones, the central zone being the area centered on the apex with a diameter of 2 mm; the second zone was an annulus between the 2 mm and 6 mm diameters; the third zone was between the 6 mm and 10 mm diameters; and the fourth zone from the 10 mm to 12 mm diameters. In addition, the cornea was also subdivided into three parts based on depth. The anterior layer was the anteriormost 120 μm, the posterior layer was the posteriormost 60 μm, and the central layer was defined as the part between these two layers. Right eyes of the participants were used for the statistical analyses. Demographic data, mean corneal power, corneal volume, anterior chamber depth, central corneal thickness, and corneal density were compared between the groups.

### Statistical Analysis

Statistical analyses were done using SPSS software (version 21.0, SPSS, Inc. Chicago, IL, USA). The results are presented as the mean ± standard error of mean (SEM). Normality of the data distribution was evaluated using the Kolmogorov-Smirnov test. Independent-samples t-test and chi-square tests were used to compare measurements between the two groups. The Pearson correlation coefficient was used to assess the strength of the correlations between corneal densitometry and simulated keratometry (Sim K) and central corneal thickness. Statistical significance was defined as a p value less than 0.05. Post-hoc calculation of statistical power was performed using NCSS-PASS software (NCSS, Utah, USA).

## RESULTS

Twenty-three consecutive RA patients and 22 age-matched healthy individuals were included. The demographic findings of the groups are presented in Table 1. There was no statistically significant difference between the RA and control groups with respect to age or gender (p=0.487 and p=0.514, respectively).

Anterior segment parameters of the groups, measured by the Pentacam system, are presented in Table 2. There were no significant differences among the groups in terms of Sim K (p=0.381), posterior K (p=0.837), corneal volume (p=0.337), or anterior chamber depth (p=0.487). Central and thinnest corneal thickness measurements of the RA patients (544.43±6.79 µm, 535.13±7.22 µm) were lower than those of the control group (554.54±6.25 µm, 547.68±6.34 µm), though the difference was statistically insignificant.

When corneal densitometry findings were compared, it was seen that total corneal densitometry was higher in the RA group (p=0.030), although there was no evident opacity. In addition, when the corneal subdivisions were evaluated, a higher density was found in the RA group in the 2 mm and 2-6 mm radial zones of the anterior layer (p=0.001 and p=0.002, respectively) and 10-12 mm zone of both the central and posterior layers (p=0.035 and p=0.018, respectively). The corneal densitometry measurements of RA patients and healthy control subjects are shown in detail in Table 3. Corneal densitometry was not significantly correlated with Sim K or central corneal thickness both in RA patients and healthy control subjects.

## DISCUSSION

Analysis of corneal densitometry has gained popularity after the introduction of the Pentacam densitometry program. The technique allows assessment of pathologies and changes in the cornea by means of a noninvasive examination that is repeatable and quick to perform. Since corneal transparency is the result of a complex organization including regular spacing of the collagen fibrils and extracellular matrix and balanced keratocyte components, high levels of corneal light backscatter may be observed even in the absence of haze or scar.^14,15^ In the present study, we measured the corneal densitometry of RA patients with clinically clear corneas and compared their results with those of age-matched healthy control subjects.

Villani et al.^16^ observed significantly higher numbers of hyperreflective stromal cells in the corneas of RA patients when compared to healthy individuals. They stated that those keratocytes were in a specific stage of metabolic activation induced by proinflammatory cytokines such as interleukin (IL)-1 and IL-6. They also demonstrated an increase in basal epithelial cells and anterior and posterior stromal cells. We hypothesized that corneal densitometry of RA patients may be altered in the absence of haze or scar due to changes in the cellular components of the cornea and subclinical inflammation.

In our study, it was seen that total corneal densitometry was statistically higher in the RA group, although there was no evident opacity or infiltration. In addition, when subdivisions of the cornea were evaluated, a higher density was found in the RA group in the 0-2 and 2-6 mm radial zones in the anterior layer.

In the central and posterior layers, a higher densitometry was also observed in the peripheral 10-12 mm annulus. But peripheral regions must be interpreted with caution in this method, as the repeatability and reproducibility are low according to previous studies.^17,18^

Reduced superficial and stromal thickness has been reported even in the absence of secondary Sjögren’s syndrome and was associated with increased proteolytic activity of the stroma and increased tangential forces on an abnormal, irregular epithelial surface.^16,19^ Cingü et al.^20^ noted lower central corneal thickness and corneal volume in RA patients, but statistically similar corneal power findings. In our study, corneal thickness in the RA group was also lower than in the control group, but the difference was statistically insignificant. This may be attributed to the low number of subjects. When other anterior segment parameters of RA patients were evaluated, it was seen that the groups were similar in terms of anterior chamber depth, corneal volume, and Sim K.

To our knowledge this is the first study to demonstrate abnormal densitometry findings in patients with RA. The reason RA patients had higher corneal densitometry values in our trial may be explained by formation of a hyperreflective stroma due to the increase in the number of activated keratocytes and subclinical inflammation in the clear cornea.

### Study Limitations

One important limitation of this study is the lack of an a priori sample size calculation. In this study, post-hoc calculation of the statistical power rather than a calculation of the sample size was performed due to the paucity of published data. Although the relatively small sizes of the groups may be a limitation, the present study is unique in measuring corneal densitometry in RA patients. A further limitation of the study is the lack of repeated lens densitometry measurements. Nevertheless, the high interobserver and intraobserver repeatability of Scheimpflug images and densitometric analyses has been demonstrated previously in the literature.21 Finally, dry eye may be a confounding factor for corneal densitometry. Since the major differences in corneal densitometry between the groups were observed in the anterior layer, it could also be attributed to dry eye.

## CONCLUSION

RA patients have significantly higher corneal densitometry values when compared to healthy control subjects. However, our results should be confirmed with further prospective studies investigating corneal densitometry in RA and other inflammatory conditions which may affect the ocular surface. Corneal densitometry as an objective measure of corneal clarity warrants further longitudinal studies in order to ascertain its clinical relevance.

## Figures and Tables

**Table 1:**
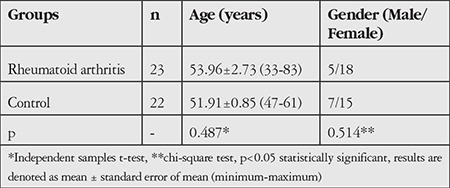
Demographic data of patients

**Table 2:**
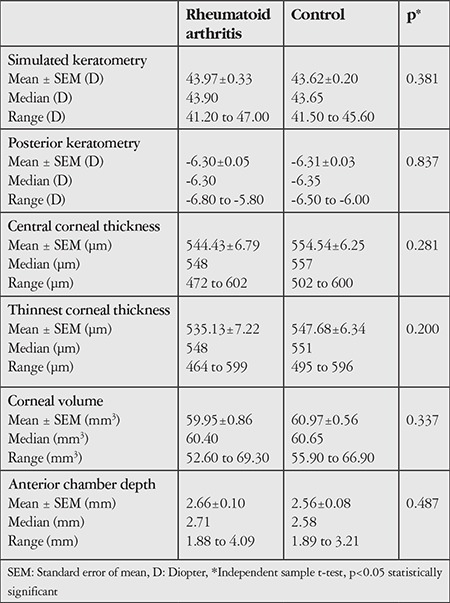
Comparison of anterior segment parameters

**Table 3:**
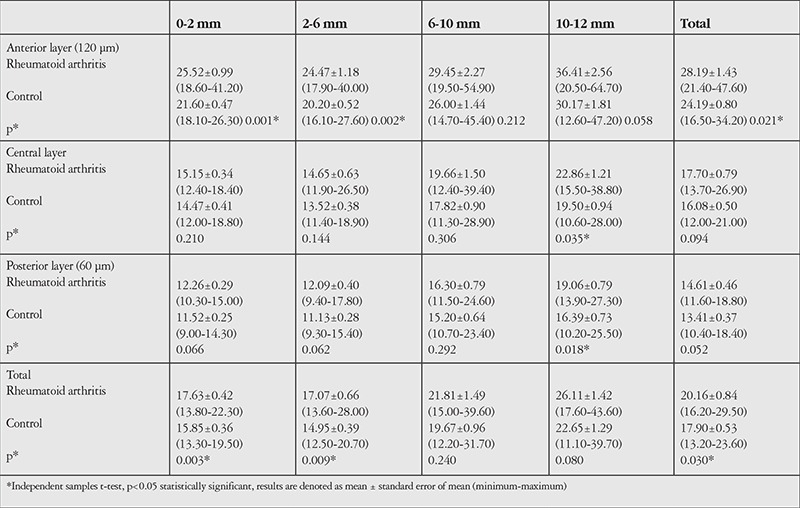
Comparison of corneal densitometry measurements
